# Adolescents expressing school massacre threats online: something to be extremely worried about?

**DOI:** 10.1186/1753-2000-6-39

**Published:** 2012-12-15

**Authors:** Nina Lindberg, Atte Oksanen, Eila Sailas, Riittakerttu Kaltiala-Heino

**Affiliations:** 1 Department of Adolescent Psychiatry, Helsinki University Central Hospital, Psykiatriakeskus, HUS, P.O. Box 590, Helsinki, 00029, Finland; 2 The Finnish Youth Research Society, Asemapäällikönkatu 1, Helsinki, 00520, Finland; 3 Kellokoski Hospital, Kellokoski, 04500, Finland; 4 Department of Adolescent Psychiatry, Tampere University Hospital, Pitkäniemi, 33380, Finland; 5 Vanha Vaasa Hospital, Vierinkiventie 1, Vaasa, 65380, Finland; 6 Medical School, University of Tampere, Tampere, 33014, Finland

**Keywords:** School massacre threat, School shootings, Adolescence, Internet, Online, Violent ideation

## Abstract

**Background:**

Peer groups identified through the Internet have played an important role in facilitating school shootings. The aim of the present study was to determine whether the adolescents who had expressed a school massacre threat online differed from those who had expressed one offline.

**Methods:**

A nationwide explorative study was conducted on a group of 77 13- to 18-year-old adolescents sent for adolescent psychiatric evaluation between November 2007 and June 2009 by their general practitioners because they had threatened to carry out a school massacre. According to the referrals and medical files, 17 adolescents expressed the threat online and 60 did so offline.

**Results:**

The adolescents who expressed their threats online were more likely to be bullied and depressed, had more often pronounced the threat with clear intention and had more often made preparations to carry out the act. In contrast, the adolescents who expressed their threats offline were more likely to have problems with impulse control and had showed delinquent behavior prior to the massacre threats.

**Conclusions:**

The Finnish adolescents who expressed their massacre threats online could be considered a riskier group than the group who expressed the threats offline. Further studies with larger sample sizes are needed to elucidate this important topic.

## Background

Youth today live their lives increasingly online. The Internet provides opportunities for self-expression, hobbies, and socializing with friends. With the rapid development of social media, adolescent peer groups have become computer-mediated, and these online groups are becoming an additional source of identity [1,2]. According to *EU Kids Online*, 82% of European youth aged 15–16 had a profile on a social networking site, such as Facebook in 2010 [3]. Because their lives become increasingly computer-mediated, these young people are also expressing various concerns via online behavior. In particular, youth who face problems offline are more likely to engage in risky online behavior [4,5].

Current research on youth and the Internet emphasizes the co-existence of opportunities and risks [3,6]. Concerns include cyberbullying and sexual harassment as well as solicitation online [7-11]. The Internet provides easy access to controversial and violent material. Visiting the “snuff” sites (portraying actual murders or deaths by people), for example, is considered a developmental risk [12]. Furthermore, various kinds of harmful or risky online communities, including pro-self-harm and pro-suicide websites, have proliferated [13-16]. Even extremely shocking and rare events such as school shootings have become objects of fascination for some youth, and the Internet makes it easier to find others with similar radical and deviant opinions [17-19].

Peer groups identified through the Internet have played an important role in facilitating school shootings. In Finland, two school shootings took place in November 2007 and September 2008 and became the most lethal mass murder cases in the criminal history of Finland. Both offenders were young males who idealized earlier American and German shooters and used the Internet to document their positive thoughts and ideas about violence as well as videos and statements about their future intentions. Both of the shooters were active in pro-school-shooting online communities, which supported their fantasies of violent revenge. Interestingly, neither of the Finnish school shooters found any encouragement for their intentions from their offline peer groups. Rather, both Finnish and international online groups did support their ideas [20].

It is possible that the online world enables the violent and suicidal ideation for some adolescents. Such behavior is more likely to occur when adolescents are already experiencing problems in their daily offline world [4,5]. Even so, one should not be underestimate the relevance of the online world. Group pressures, for example, may be even stronger in a computer-mediated setting than face-to-face [21]. Thus, deviant online communities, such as pro-school-shooting groups, may be able to reinforce the violent ideation. The Internet and social media mostly serve as tools; finding other like-minded people is easier in the online world.

Because school shooters have called themselves “rebels”, they have become objects of admiration for some young people, especially after the Columbine shootings in the United States in 1999 [22-24]. School shooters have cultivated images of martyrdom and political revolt against oppressors [20]. Such romanticized images may make school shooters rebels in the eyes of young people troubled by experiences of bullying at school and with psychological problems which magnify the seriousness of these experiences. For some young people, school shooters have become heroes, and authors have speculated that committing a school shooting threat in particular becomes a way to show sympathy and admiration for the school shootings [25]. After dramatic violence, threats of similar acts are likely to occur. Threats peaked, for example, after both the Columbine massacre in the US (1999) [26] and the Winnenden tragedy in Germany (2009) [27]. The Internet likely facilitates the expression of such acts.

Before the Jokela school shooting tragedy in November 2007, threats to carry out a massacre warranting a police investigation were rare, with about 5–10 threats per year, as continues to occur in the other Nordic countries. Immediately after the school shooting in November 2007, 87 police registered threats, and within a few weeks of the second school shooting in September 2008, more than a hundred new threats were communicated. Still, in 2011, the annual number of threat was nearly 60 cases (police statistics, personal communication from Savolainen, M.).

Referrals to adolescent psychiatric services due to threats of school massacres peaked in Finland after the Jokela and Kauhajoki school shootings. Through the chief adolescent psychiatrists responsible for specialist-level adolescent psychiatric services in the different hospital districts in Finland, we identified all 77 adolescents aged 13–18 who underwent adolescent psychiatric assessment for having expressed threats of a school massacre after the two incidents [28]. Of them, 17 had expressed the massacre threat online.

In the field of adolescent psychiatry, studies focusing on links between the Internet, adolescent mental health problems and antisocial behavior remain scarce. The aim of the present study was to determine whether the Finnish adolescents who had expressed a school massacre threat online differed from those who had expressed one offline. The focus was on current psychiatric symptoms and clinical diagnoses as well as on adverse family life events, mental health contacts, academic performance and delinquency prior to the threat. We investigated any adolescents` experiences of being bullied at school prior to the threat. We also evaluated the characteristics of the index threat as well as the risk posed by the adolescent.

## Method

### Participants

We conducted a nationwide study on a group of 13- to 18-year-old adolescents sent for adolescent psychiatric evaluation between November 2007 and June 2009 by their general practitioners due to having threatened to carry out a school massacre.

Information about the study was sent via both e-mail and post to all the chief physicians in the field of adolescent psychiatry in Finland. The chief physicians were asked to go through the referrals made between November 2007 and June 2009 and to select referrals that included threats to carry out a school massacre. The chief adolescent psychiatrists were asked to send the patients` identifying information (names and social security numbers) in registered letters to the researchers. The researchers (N.L. and R.K-H.) then traveled to the adolescent psychiatric units and studied the medical files of the index adolescents.

The study comprised a total of 77 adolescents (67 boys and 10 girls) with a mean age of 15.0 years (SD 1.48, range 13–18 years) (for details; see [28]). According to the referrals and medical files, 17 (22%) adolescents expressed the threat online and 60 (78%) did so offline. The offline threats were expressed orally to a teacher, therapist or school friend in 43 cases and verbally in a letter, essay or exam paper in 17 cases. Those first informed then initiated the process that resulted in the threatener undergoing examination by the general practitioner, who then referred him or her to adolescent psychiatry. The online threateners were again identified when their peers or the police noticed the threat online and initiated the process that resulted in the threatener receiving a medical consultation, and later undergoing adolescent psychiatric evaluation.

### Variables

Data on the adolescents` psychiatric symptoms were collected from the referral and the medical charts written during the assessment and treatment initiated after the threat was expressed (for details; see [28]). A total of 21 core symptoms of the adolescents (see Table 1) were recorded with a checklist (yes/no), which was originally developed as a screening tool for clinical situations and has served in research that applies retrospective data collection from medical charts [29].

**Table 1:** The comparisons of the adolescents who expressed the massacre threat online (n =17) and those who expressed it offline (n = 60)

	**Internet**	**Other ways**	**Statistics**	**p**	**Phi**
**Gender:** girl	3/17 (18%)	7/6 (12%)	#	0.282	0.06
**Stressful life event/s in family during the 6 months prior to the index threat**	11/17 (65%)	33/60 (55%)	0.510	0.475	0.08
**Client of child welfare prior to the index threat**	2/17 (12%)	19/60 (32%)	#	0.104	0.19
**Performing well or average academically**	10/17 (59%)	41/60 (68%)	0.536	0.464	0.08
**Mental health contact prior to the index threat**	9/17 (53%)	35/60 (58%)	0.157	0.692	0.05
**Current symptoms in the adolescent psychiatric evaluation**					
Symptoms of anxiety	14/17 (82%)	36/60 (60%)	2.907	0.088	0.20
Depressive symptoms	16/17 (94%)	32/60 (53%)	9.385	0.002*	0.35
Problems with impulse control	6/17 (35%)	40/60 (67%)	5.421	0.020*	0.27
Aggressive outbursts/tantrums	9/17 (53%)	31/60 (52%)	0.009	0.926	0.01
Suicidal ideation	9/17 (53%)	26/60 (43%)	0.493	0.483	0.08
Destroying property	5/17 (29%)	27/60 (45%)	#	0.028*	0.13
Non-physical aggression towards others	5/17 (29%)	26/60 (43%)	#	0.404	0.12
Violence against others	6/17 (35%)	25/60 (42%)	0.224	0.636	0.05
Harmful use of alcohol	4/17 (24%)	15/60 (25%)	#	1.000	0.01
Isolation	7/17 (41%)	12/60 (20%)	3.196	0.074	0.24
Psychotic symptoms	3/17 (18%)	15/60 (25%)	#	0.748	0.07
Attention problems	5/17 (29%)	25/60 (42%)	#	0.040*	0.20
Use of illicit drugs	4/17 (24%)	2/60 (3%)	#	0.019*	0.31
Running away	1/17 (6%)	5/60 (8%)	#	0.551	0.08
Suicide attempt	0/17 (0%)	2/60 (3%)	#	1.000	0.09
Truancy/school refusal	3/17 (18%)	6/60 (10%)	#	0.150	0.12
Self-harming behavior	0/17 (0%)	0/60 (0%)			
Manic behavior	0/17 (0%)	1/60 (2%)	#	1.000	0.06
Symptoms of eating disorders	0/17 (0%)	6/60 (10%)	#	0.329	0.16
Inappropriate sexual behavior	0/17 (0%)	0/60 (0%)			
**Victim of bullying at school**	12/17 (71%)	18/60 (30%)	9.276	0.002*	0.35
**Primary clinical diagnoses**					
F0-09	0/17 (0%)	0/60 (0%)			
F10-19	0/17 (0%)	0/60 (0%)			
F20-29	1/17 (6%)	8/60 (13%)	#	0.674	0.10
F30-39	4/17 (24%)	12/60 (20%)	#	0.743	0.04
F40-49	2/17 (12%)	6/60 (10%)	#	1.000	0.02
F50-59	0/17 (0%)	2/60 (3%)	#	1.000	0.09
F60-69	2/17 (12%)	1/60 (2%)	#	0.121	0.22
F70-79	0/17 (0%)	1/60 (2 %)	#	1.000	0.06
F80-89	5/17 (29%)	8/60 (13%)	#	0.146	0.18
F90-98	3/17 (18%)	15/60 (25%)	#	0.785	0.07
**No psychiatric diagnosis**	0/17 (0%)	6/60 (10%)	#	0.329	0.16
**Previous delinquency**					
Yes	1/16 (6%)	20/50 (40%)	#	0.013*	0.31
**A = Attitudes**					
Positive attitudes towards aggressive behavior in general	13/14 (93%)	45/51 (88%)	0.244	0.621	0.06
Positive attitudes against previous school shootings	12/15 (86%)	31/47 (65%)	1.055	0.304	0.13
Felt justification for the attack	12/12 (100%)	46/50 (92%)	1.026	0.311	0.13
**Motive**					
Revenge	9/17 (53%)	25/58 (43%)	0.513	0.474	0.08
Anger and hatred in general	5/17 (29%)	22/58 (38%)	#	0.579	0.07
Desire to die (homicide-suicide)	5/17 (29%)	19/58 (33%)	#	1.000	0.03
Joke	0/17 (0%)	3/58 (5%)	#	1.000	0.11
Wanting attention	1/17 (6%)	3/58 (5%)	#	1.000	0.01
**C = Capacity to fulfill the threat**					
Yes	10/12 (83%)	32/47 (68%)	1.084	0.298	0.14
**T = Thresholds crossed**					
Preparations made	9/16 (56%)	11/50 (22%)	6.732	0.009*	0.31
**I = Intention of the threat**					
Clear intention to commit the act	6/13 (46%)	8/57 (14%)	6.825	0.009*	0.31
**O = Others` reactions**					
Parents took the threat seriously	9/16 (56%)	29/59 (49%)	0.254	0.615	0.06
**N = Non-compliance to risk reaction**					
The adolescent was against psychiatric evaluation/treatment	6/17 (35%)	38/60 (53%)	4.253	0.039*	0.24

The chi-square test or Fisher’s exact test (#) were used to compare the groups.

^*^ = statistically significant.

With the help of a structured checklist [29], adverse family life event/s (domestic violence, parental mental disorders, parental substance abuse problems, divorce or separation processes, severe somatic illness of parent/s, severe financial difficulties, severe problems related to sisters, [suspected] sexual abuse, bereavement) from the last six months before the index threat were recorded. Patients` main psychiatric diagnoses (F0-09 Organic, including symptomatic, mental disorders; F10-19 Mental and behavioral disorders due to psychoactive substance use; F20-29 Schizophrenia, schizotypal and delusional disorders; F30-39 Mood disorders; F40-49 Neurotic, stress-related and somatoform disorders; F50-59 Behavioral syndromes associated with physiological disturbances and physical factors; F60-69 Disorders of adult personality and behavior; F70-79 Mental retardation; F80-89 Disorders of psychological development; F90-98 Behavioral and emotional disorders) were collected as given at discharge by the treating psychiatrist according to the ICD-10 [30]. Delinquency, academic performance, mental health contact, social welfare contact and whether he or she was a victim of bullying prior to the index threat were collected from the medical files.

We evaluated the characteristics of the threat expressed and the risk posed by the adolescent in question using an approach presented by Borum and Reddy [31]. In their model, ACTION, a mnemonic guide helps the evaluator to consider the relevant aspects of the motivation, behavior and attitudes of the potential perpetrator in order to advise for interventions. All available information from the person expressing the threats, the informants close to him or her, and their medical files can be used. In the ACTION mnemonic guide, A (Attitudes) refers to positive attitudes towards violence and the embrace of violence-positive ideologies as well as the perception that violence is justified in one’s own situation (in the present data, for example, the adolescent expressed positive feelings towards Nazism, fascism, terrorism, etc.). C (Capacity) refers to the physical and cognitive abilities of the potential perpetrator and his or her potential to fulfill the expressed threat. In T (Thresholds crossed), the evaluator considers whether the person has made preparations to fulfill the threat (i.e., the adolescent told that he or she bought a gun, practiced shooting, made a bomb, drew a map of how to attack the school building, etc. in order to carry out the school massacre). In particular, preparations that are in themselves illegal should be considered as signs of increased risk. I (Intent), assesses: Is the person passively attracted to the thought of violence (the adolescent was attracted to the idea of a school massacre, but described no plans to perform it) or actively planning an act (the adolescent revealed a plan for how to perform the massacre)? O (Others’ reactions) refers to how those close to the person react to the threats expressed. This includes whether they believe that the person could actually commit the act that he or she is threatening as well as also any admiration and support actually expressed or believed to be expressed for the potential perpetrator. N (non-compliance with risk reduction) assesses whether the person is motivated to accept interventions that help him or her avoid violence. The approach is neither a structured scale with fixed response alternatives that produce a score nor a rigorously structured, validated violence risk assessment tool, but an aid that helps to systematically consider qualitative information, which serves as a basis for evaluating the risk and choosing interventions most likely to reduce the risk. The conclusions are made clinically after reviewing relevant aspects with the help of the mnemonic guide.

The study was approved by the Ethics committee of the Helsinki University Hospital and the Ministry of Social Affairs and Health. The Ministry of Social Affairs and Health may allow researchers to assess patient/client files created in health and social care if the research is appropriately motivated and has received the approval of the ethics committee and if data security issues are arranged appropriately. Thus, the recruited health/social care institutions choose their own procedures regarding whether they will allow the study to use their own registers. Privacy protections vary between countries, but all personally identifiable information remained secure.

### Statistics

We conducted data analyses with the SPSS 11.0.1 statistical software package. We used the independent samples *t*-test, the chi-square test and the Fisher`s exact test to compare the groups. The findings were considered significant when p < 0.05. The magnitudes of effect size phi were interpreted as follows: 0.00 to under 0.10 = negligible association, 0.10 to under 0.20 = weak association, 0.20 to under 0.40 = moderate association, 0.40 to under 0.60 = relatively strong association, 0.60 to under 0.80 = strong association and 0.80 to 1.00 = very strong association [32].

## Results

For the results, see Table 1. The mean age of the adolescents who had expressed the threat online did not significantly differ from the mean age of the adolescents who had expressed the threat offline (online threateners: mean =14.9 years, sd ± 1.45, offline threateners: mean =15.0 years, sd ±1.50, df = 75, t = −0.334, p = 0.741). The adolescents who had expressed the threat online showed more often depressive symptoms and use of illicit drugs than the adolescents who had expressed the threat offline (effect sizes showed moderate associations). They more often expressed feelings of being a victim of bullying at school (moderate association). They also more often expressed a clear intention to carry out a school massacre and had more often made preparations for an attack than did the offline threateners (moderate associations). The adolescents who had expressed the threat offline more often exhibited problems with impulse control (weak association), destroying property (weak association), attention problems (moderate association) and previous delinquency (moderate association) than did those who expressed the threat online. The online threateners more often expressed positive feelings towards psychiatric evaluation/treatment than did the offline threateners (moderate association).

## Discussion

To the best of our knowledge, this is the first study to compare adolescents committing school massacre threats online to those who had threatened to do so offline. The main finding was that adolescents who had expressed school massacre threats online were more likely to report being bullied and depressed with no previous delinquency had more often issued the threat with clear intention and had more often made preparations to carry out the act. In contrast, the adolescents who had expressed the threat offline were more likely to have problems with impulse control and to had shown delinquent behavior prior to the massacre threats. The literature describes such a group of impulsive and aggressive young people as low risk [27]. These impulsive and aggressive adolescents exhibited previous delinquency and the massacre threat was expressed spontaneously in a face-to-face conflict situation; they had no serious plan or intention to carry out their threat.

Our results indicate that adolescents who expressed their massacre threats online could be considered a far riskier group than the group who expressed their treats offline. Especially crucial is that half of them had made preparations to carry out the threat, which is considered a crucial step in the process of becoming a school shooter. It is important to emphasize that the school shooters do not act spontaneously, but rather plan their actions carefully in a long developmental process from idea to plan and to acting on that plan [22,33]. Depression is known to be another risk factor in this developmental process [22,33-36]. On the other hand, those adolescents who make even one-time threats in person to their teachers, therapists or in essays/exam papers may be more easily identified for assessment than those who issue one-time threats online. Thus, consequently, the distinguishing factor, rather than whether the adolescents expressed their massacre threats online versus offline, is the degree or length of obsession with and communication about massacre threats.

More than two thirds of the online threateners reported being victims of bullying. This experience was much more common among the online threateners than among those expressing threats offline, and was also more common among both groups than among teenagers at large, of whom 10-20% report being bullied regularly [37-39]. Bullying is defined as repeated, intentionally aggressive behavior towards another person in a situation where a power imbalance renders the victim weaker than the bully/bullies [40]. Bullying is also a group process involving not only the bully and the victim, but also a majority of the children/adolescents, who relate to the bully and victim and reinforce/facilitate the behavior of the bully/bullies, or, sometimes, defend the victim [41]. Thus, a victim of bullying may not only repeatedly experience situations of abuse, humiliation and frustration, that may provoke helpless anger and depression and give way to thoughts of violence, but may also feel generally excluded and rejected by a majority of his or her peers. Peer rejection is known to be a risk factor for violence among adolescents [42]. Mental disorders such as depression, social anxiety or pathological personality development may also result in a distortion of the processing of social information: an adolescent suffering from mental disorders producing a negative self-perception or paranoid position towards others may perceive others behaving in a rejective or hostile manner, even in social interactions that others intend to be neutral or even positive [43-45]. This may also lead to isolation, a symptom that was borderline significantly more common among online threateners than among other threateners, and more intensive submersion into the online world, where at-risk adolescents can find support for violent fantasies. Isolation has been identified as an essential step in the process of becoming a school shooter [22,33]. Our data do not allow us to verify what kinds of interactions objectively took place between the threateners and their peers. However, an adolescent is likely to react predictably to perceived circumstances. Interventions to reduce bullying are important, but individually tailored mental health interventions are also needed among those who self-report being victims of bullying to stop the adolescent from proceeding along the pathway towards aggressive outbursts. It is noteworthy that the online threateners in our sample actually had a more positive attitude towards psychiatric evaluation than did those who expressed their threats offline. This suggests that even though they appeared to be the most seriously potentially dangerous adolescents in our sample, they may also have certain insight into their own pathological development; psychiatric intervention may therefore prove beneficial. For this reason, it is particularly important to arrange psychiatric evaluations for teenagers who express massacre threats via the Internet.

The strength of the present study was its nationwide scope. Nevertheless, it has its limitations. First, the diagnoses were based not on structured interviews, but were taken from medical files. In this regard, however, the basic diagnostic procedures in Finland have proved reliable [46-48]. Second, the quality of the medical files varied, and the data on family life, school and previous criminality was based on information provided by the adolescents and their guardians, teachers and social workers. The official files from child welfare services or criminal records (note: in Finland, the minimum age for criminal liability is 15 years) were not used. Third, variables that are typically investigated in Finnish adolescent psychiatric examination were collected. The items in the checklists are symptoms and events that Finnish adolescent psychiatry considers important to an adolescent’s well-being and are most likely assessed in routine adolescent psychiatric assessment. Occasionally, however, an event may have occurred or a symptom may be present even if it is not recorded in the adolescent`s case history. Because of this, the psychopathology found in the present sample is more likely underestimated than overestimated. Fourth, the number of online threats remained small, so this study must be regarded as preliminary. Fifth, the observed effect sizes were not large, whereas most effect sizes were moderate. Sixth, although determining the total number of school massacre threats expressed during the study period proved impossible, estimates by the Finnish police this number is in hundreds. It is likely that the sample of the present study consisted of those adolescents of whom the school and social authorities had been the most concerned regarding their mental health. Because of this, generalizing the results to other threateners must be done with caution. Also, the raters did not double-rate. However, the items were concrete, leaving little room for interpretation. It is also worth noting that some of those who post threats online are never identified and can thus not be studied with approaches such as those used in the present study. The findings and conclusions of the present study are exploratory, and further studies with larger sample sizes are needed to elucidate this important topic.

## Conclusion

The Finnish adolescents who expressed their massacre threats online could be considered a riskier group than the group who expressed the threats offline.

## Competing interests

The authors declare no conflict of interest.

## Authors’ contributions

NL reviewed the medical files, organized the data and served as the first author. AO participated in the writing process. ES analyzed the data and participated in the writing process. RK-H reviewed the medical files and participated in the writing process. All authors read and approved the final manuscript.
